# Post-keratoplasty astigmatism management by relaxing incisions: a systematic review

**DOI:** 10.1186/s40662-017-0093-7

**Published:** 2017-12-06

**Authors:** Gaëlle Ho Wang Yin, Louis Hoffart

**Affiliations:** 1 Ophthalmology Department, Aix-Marseille University - APHM, Hôpital de la Timone, 264 rue Saint Pierre, 13 385 Marseille Cedex 5, France; 2 Institut Fresnel UMR 7249, Aix Marseille Université, CNRS, Centrale Marseille, Domaine universitaire de Saint-Jérôme Avenue Escadrille Normandie Niemen, 13397 Marseille cedex 20, France; 3 CERIMED, Aix-Marseille University, 27 Boulevard Jean-Moulin, 13385 Marseille cedex 05, France; 4 Ramsay Générale de Santé, Clinique Monticelli-Velodrome, Marseille, France

**Keywords:** Penetrating keratoplasty, Astigmatic keratotomy, Relaxing incisions, Femtosecond laser

## Abstract

Postoperative visual acuity can be limited by post-keratoplasty astigmatism, even with a clear corneal graft. Astigmatism management can be performed by selective suture removal, adjustment of sutures, optical correction, photorefractive procedures, wedge resection, intra-ocular lens implantation, intracorneal ring segments, relaxing incisions with or without compression sutures and repeated keratoplasty. Relaxing incisions can be made in the graft, graft-host interface or host cornea. Despite the unpredictability of the method because the flat and steep meridians are usually not orthogonal after penetrating keratoplasty, with asymmetric power distribution, all the studies showed an overall reduction of refractive, keratometric or topographic astigmatism, ranging from 30% to 72% with manual or femtosecond-assisted techniques. Most patients with astigmatism higher than 6 diopters had residual cylinder less than or equal to 3 diopters, which can be treated by laser excimer ablation or secondary intraocular lens implantation.

## Background

The aim of penetrating keratoplasty (PK) is to improve a patient’s postoperative vision. Despite a clear corneal graft, visual acuity can be limited by astigmatism [1]. Based on several studies, 15-20% of patients may develop 5 or more diopters (D) of post-keratoplasty astigmatism [2–5].

Post-keratoplasty astigmatism management can be done before or after suture removal. If done before, it is called suture-in post-keratoplasty astigmatism. In this case, topography-guided suture manipulation, including selective suture removal [6] guided by corneal topography [7], or adjustment of sutures along the steep meridian of astigmatism or adjustment of running sutures are key factors for controlling astigmatism.

After suture removal, post-keratoplasty astigmatism can be managed by optical correction such as contact lenses or glasses [8], which can be compromised by irregular astigmatism associated with high-order aberrations [9]. Other astigmatism managements include incisional keratotomy in the graft [10, 11] or host cornea [12], and photorefractive procedures such as photorefractive keratectomy (PRK), laser in situ keratomileusis (LASIK) or topography-guided excimer laser ablation [13–16], compression sutures and a combination of relaxing incisions and compression sutures (augmented relaxing incisions) [17–19]. Wedge resection [20], intra-ocular lens (IOL) implantation [21] or intra-corneal ring segments (ICRS) [22] can also be considered. As a last resort, repeated keratoplasty [23] may be necessary.

The purpose of this review is to provide surgeon updated information about post-keratoplasty astigmatism management with arcuate keratotomy and to help define a strategy for the correction of this post-keratoplasty astigmatism. Questions we intend to answer were: Is femtosecond laser-assisted relaxing incision better than conventional relaxing incision for the management of post-penetrating keratoplasty astigmatism? Which nomogram and which length and depth should we use for the correction?

## Main text

### Search strategy

A review on PubMed and Cochrane was performed, analyzing all the publications from 1986 to 2017, concerning the topic of post-keratoplasty astigmatism management with keratotomies (keywords: arcuate keratotomy, astigmatic keratotomy, penetrating keratoplasty, post-keratoplasty astigmatism). Clinical trials, systematic review, non-randomized prospective study and case series of more than 6 patients, published in an English language journal with an impact factor greater than or equal to 2.0 were reviewed.

### General considerations

The corneal graft-host junction generally heals by 1 year after transplantation and corneal surface stability is generally achieved by 3 to 4 months after complete suture removal [24]. Before any surgical astigmatism management, a complete ophthalmologic examination including manifest and objective refraction, keratometry, and slit biomicroscopy should be performed to evaluate the graft-host interface and clarity of the graft, corneal topography, pachymetry, specular microscopy and wave-front analysis.

### Etiology of astigmatism

Factors influencing post-penetrating keratoplasty astigmatism involve the donor tissue and the host.

Infant donor corneas induce greater astigmatism than adult donor tissues [25]. Non-uniform peripheral changes in the donor tissue can affect the apposition and healing between the host and donor tissue [26].

The severity of the underlying disorder (such as keratoconus, keratoglobus or pellucid marginal degeneration) [27] also plays a role in post-penetrating keratoplasty astigmatism.

During surgery, trephination (oval or eccentric) [28]; graft size (large graft diameters induce less astigmatism but are associated with a high risk for vascularization and allograft rejection, small grafts are associated with larger amounts of astigmatism) [29]; corneal thickness and donor-recipient disparity [30]; a poor suturing technique (single or double running sutures, interrupted sutures, all 3 techniques are comparable) [31] are factors involved in post-keratoplasty astigmatism. Time of suture removal or adjustment [32–34] is also important.

Post-operative drugs, inflammation, corneal vascularization, rejection and wound dehiscence affect wound healing and may be associated with astigmatic changes.

### Principles of relaxing incisions

Relaxing incisions can be made for patients with keratometric astigmatism, 3 to 4 months after complete suture removal [24]. The main principle of relaxing incisions is to flatten the steep corneal meridian by one or two incisions perpendicular to it. This flattens the steepest meridian with reciprocal steepening of the meridian 90° away, this effect is known as “coupling effect” [35]. Arc length is related to the coupling ratio: a coupling ratio of close to 1.0 is obtained with 30–90° incisions, which should not change the resulting spherical equivalent. Incisions less than 20° arc length have a coupling ratio greater than 1.0 while those greater than 100° have a coupling ratio less than 1.0. Relaxing incisions are made under topical anesthesia, on both sides of the steepest meridian, usually, with an arc length of 45° to 90°, and can be done in the 7.0 mm optical zone for maximal effect. The site and length are topography-guided [36]. In arcuate incisions, each incision is at the same distance from the visual axis to avoid uneven distribution of the force on the corneal architecture and allows rapid visual rehabilitation. Arcuate keratotomy can be made with or without 10-0 nylon compression sutures, these are added to achieve overcorrection of astigmatism in the opposite meridian (90° away). Selective suture removal is initiated 3 to 4 weeks later.

The site of arcuate keratotomy can either be in the donor cornea or at the graft-host interface, or in the host cornea [12]. Incisions in the recipient corneas are not recommended as it is believed that the scarring at the graft-host junction changes the biomechanical state of the cornea. The keratoplasty wound is supposed to form a new limbus, blocking the effect of relaxing incisions in the recipient cornea [37].

Relaxing incisions can be made manually (freehand with a diamond knife [35] or mechanized with Hanna arcitome [38]) or with femtosecond laser technology [39].

### Nomograms

The optical zone, arc length and depth are determined using nomograms. The effect of astigmatic correction with astigmatic keratotomy increases with greater incision depth, more central placement of the incisions, increasing patient age [40] and longer arc length.

Nomograms used on patients with congenital astigmatisms, such as the Lindstrom nomogram [40] do not apply to post-penetrating keratoplasty patients. The astigmatic effect of the incision is proportional to the preoperative cylinder [35]. Typically, post-penetrating keratoplasty arcuate keratotomy is made to a depth of 75% corneal thickness, and incisions are made at an optical zone 1.0 mm central to the graft-host junction.

The Hanna nomogram is one of the most widely used nomogram [38]. It is based on refractive astigmatism, from 2.50 D to 15 D. The arcuate keratotomies are made with an optical zone diameter ranging from 6.00–6.75 mm, with an incision depth of 75% of corneal pachymetry and an angular length from 60 to 80°.

The Nordan nomogram [41] is used by surgeons to create paired symmetric incisions, 75–85% depth of the thinnest measurement of the graft, and centered on the steepest meridian axis as follows:

- 1.75–2.5 D of cylinder with 50° arc length,
- 2.75–3.3 D of cylinder with 57° arc length,
- 3.75–4.5 D of cylinder with 60° arc length,
- 5 D of astigmatism with 70° arc length.

Another nomogram, for beveled incisions, was described by Cleary et al. [42]. These incisions, made at a 135° angle centered on the axis of the astigmatism, allowed for the anterior cornea to slide forward, decreasing wound gape. Moreover, the beveled incisions can be made at a 65–75% depth rather than 90%, with a reported comparable reduction in astigmatism versus traditional femtosecond laser-assisted arcuate keratotomy (FSAK) incisions. Arc length at 8.0 mm optical zone ranges from 60° to 85°.

The most recent nomogram was developed by Saint-Clair [43]. It is a new nomogram for femtosecond laser astigmatic keratotomy for astigmatism after keratoplasty, considering a variety of incision-related factors and the degree of pre-existing astigmatism. Incision depth, arc length and optical zone diameter depend on the pre-operative difference between the steepest and the flattest keratometry values.

### Manual versus femtosecond laser-assisted arcuate keratotomy

Astigmatic keratotomy can be performed manually using a handheld, fixed or adjustable depth diamond knife, or mechanized like the Hanna arcitome. The diamond blade is adjusted according to the proper nomogram.

FSAK improve arc length, depth and location precision compared with manual and mechanized incision [39, 44]. The surgeon can adjust location, depth and centration of the incision using the intraoperative OCT of the femtosecond laser platform.

FSAK is also associated with a lower risk of wound dehiscence, epithelial down growth, infection, and perforation or full-thickness corneal incisions [39]. Main results are summarized further.

### Other techniques

#### Intrastromal astigmatic keratotomy

Intrastromal astigmatic keratotomy (ISAK) was introduced for the treatment of low astigmatism. These incisions are not opened anteriorly, decreasing the risk of epithelial disruption, infection or wound dehiscence. ISAK was successful for naturally occurring and post-cataract surgery astigmatism [45] and residual astigmatism after refractive surgery [46].

Viswanathan & Kumar [47] treated a young keratoconic patient with over 11 D of astigmatism in both eyes after suture removal from bilateral penetrating keratoplasties. Paired ISAK with a depth of 90%, 60 μm anteriorly at a 90° side cut was made. At 4 months follow-up, keratometric astigmatism was 4.1 D in the right eye and 1.12 D in the left eye, with a corresponding reduction of 65.5% and 89.42%, respectively.

Wetterstrand et al. [48] reported the results of intra-stromal relaxing incisions after penetrating keratoplasty of 16 patients. The incisions were made intra-stromally with a length of 90°, depth of 90% of corneal thickness, diameter zone of 6.0–7.0 mm, and a safe zone of 90 μm anteriorly. Refractive cylinders decreased from 6.8 ± 2.2 D to 3.7 ± 1.7 D at 3 months follow-up, with only one bulge in the temporal incision of one patient, treated with compression suture. No other adverse effect was found. This technique seems to be relatively safe and effective.

#### DIAKIK

Loriaut et al. [49] described a novel technique, combining deep ISAK performed under a LASIK flap, for the treatment of high natural occurring and post-keratoplasty astigmatism: deep intrastromal arcuate keratotomy with in situ keratomileusis (DIAKIK).

The first step is ISAK made at a depth of 75% in eyes with penetrating keratoplasty. A LASIK flap of 100 μm was then made, lifted and ISAK were opened and the flap replaced. Laser excimer ablation can be made 1 month after for the correction of residual ametropia, after reopening of the flap.

This technique was performed in 9 patients. The mean preoperative refractive cylinder was 6.11 ± 2.54 D, and the post-operative cylinder was 2.85 ± 1.31 D. No complications were reported. This technique affords advantages of ISAK with a greater astigmatism correction, but there are potential complications of the LASIK itself.

## Results

Main results are summarized in Table 1.

**Table 1:** Main results studies

Authors	N	Study type	Treatment		Location	Refractive (R) or keratometric (K) cylinder			Complications
						Pre-op (D)	Post-op (D)	Reduction (%)	
Krachmer & Fenzl, 1980 [52]	16	Retrospective, comparative case series	Manual paired relaxing incision Depth = 50% to 66% CCT Length = 60° Graft-host interface		Graft-host interface	10.11 (K)	5.79 (K)	43 (K)	2 perforations
Price & Whitson, 1991 [55]	111	Retrospective case series	Manual paired relaxing incision Depth and length variable		Graft or graft-host interface	6.18 ± 1.56 (R)	3.34 ± 1.96 (R)	46 (R)	12 wound dehiscences
Hjortdal & Ehlers, 1998 [56]	21	Nonrandomized retrospective study	Manual relaxing incision Depth = central corneal thickness Length = 45°; 5 or 6 mm from optical zone		Graft	7 (R)	3.25 (R)	54 (R)	None
Hannush et al., 1998 [57]	29	Retrospective case series	Manual paired relaxing incision Depth = 75% CCT Length = not available 0.5 mm inside the graft-host interface		Graft	8.8 ± 3.1 (K)	3.2 ± 3.0 (K)	64 (K)	None
Borderie et al., 1999 [58]	22	Retrospective case series	Manual paired relaxing incision with Hanna arcitome Depth = 75% thinnest pachymetry Length = 60° to 80° 6 to 6.75 mm zone diameter (Hanna nomogram [38])		Graft	6.94 ± 2.11 (R)	3.85 ± 1.95 (R)	44 (R)	1 perforation, 1 overcorrection (same patient)
						9.02 ± 2.67 (K)	3.94 ± 2.19 (K)	56 (K)	
Koay et al., 2000 [59]	34	Nonrandomized retrospective study	Manual relaxing incision and compression sutures Depth = 480 μm Length = 45°		Graft-host interface	9.69 ± 3.51 (R)	3.92 ± 2.16 (R)	59.5 (R)	1 graft rejection
						9.14 ± 4.38 (K)	3.59 ± 1.92 (K)	60.7 (K)	
Wilkins et al., 2005 [35]	20	Nonrandomized retrospective study	Manual relaxing incision Depth = 600 μm Length = 60°; 6 mm from optical zone		Host	10.99 ± 4.26 (R)	3.33 ± 2.18 (R)	72 (R)	None
Bochmann & Schipper, 2006 [12]	11	Nonrandomized retrospective study	Manual relaxing incision Depth = 80% of peripheral corneal thickness Length = 30° to 90° 9 to 10 mm zone diameter		Host	6.1 ± 2.5 (R)	3.3 ± 0.7 (R)	45.9 (R)	None
						9.02 ± 3.54 (K)	3.41 ± 1.21 (K)	62.2 (K)	
Geggel 2006, [50]	26	Nonrandomized retrospective observational case series	Manual relaxing incision Depth = 80% of the thinnest pachymetry on the meridian Length = 35° to 90° 0.5 mm or 1 mm inside the graft-host junction if >10 D		Graft	8.7 ± 2.4 (K)	3.25 ± 1.74 (K)	63 (K)	None
Poole & Ficker, 2006 [60]	39	Nonrandomized retrospective study	Manual relaxing incision Depth = 90% CCT Length = 45° to 90°, 0.5 mm inside the graft-host interface		Graft	9.13 (R)	4.85 (R)	47 (R)	1 perforation
Hoffart et al., 2007 [61]	40	Nonrandomized retrospective study	Manual relaxing incision Depth = 75% CCT, length = 60° to 80° 6 to 6.75 mm zone diameter (Hanna nomogram [38])		Graft	8.84 ± 3 (R)	4.88 ± 2.5 (R)	45 (R)	1 perforation, 2 rejections
Bahar et al., 2008 [62]	40	Retrospective, comparative case series	FS: Depth = 90% CCT Length = 60° to 90° 0.5 mm inside the graft-host junction	Manual: Depth = 500 μm Length = 45° to 90° 0.5 mm inside the graft-host junction	Graft	FS: 7.84 ± 2.35 (K)	FS: 3.58 ± 2.21 (K)	FS: 54 (K)	FS: 5 overcorrections, 1 rejection
						Manual: 7.80 ± 3.14 (K)	Manual: 4.58 ± 2.95 (K)	Manual: 41 (K)	3 perforations,6 overcorrections, 1 infection
Buzzonetti et al., 2009 [63]	9	Prospective non- comparative study	FS, paired symmetric incisions 90° angled Depth = 80% thinnest pachymetry Length = 70° Optical zone = 4.8 to 6.8 mm		Graft	9.10 ± 3.90 (R)	3.10 ± 1.50 (R)	66 (R)	None
						9.80 ± 1.90 (K)	5.20 ± 1.50 (K)	47 (K)	
Nubile et al., 2009 [64]	12	Prospective noncomparative interventional case series.	FS, paired symmetric incisions 90° angled Depth = 90% pachymetry on incision location Length = 40° to 80 ° 1 mm inside the graft-host junction		Graft	7.16 ± 3.07 (R)	2.39 ± 1.62 (R)	66 (R)	2 perforations
Hoffart et al., 2009 [39]	20	Prospective comparative randomized study	FS or Hanna arcitome Depth = 75% thinnest pachymetry Length = 60° to 80° 6 to 6.75 mm zone diameter (Hanna nomogram [38])		Graft	FS: 8.6 ± 3 (R)	FS: 3.9 ± 2.4 (R)	FS: 55 (R)	1 perforation
						Hanna: 6.7 ± 2.1 (R)	Hanna: 4.7 ± 2.4 (R)	Hanna: 30 (R)	
Kumar et al. 2010 [65]	34	Retrospective case series	FS, paired symmetric incisions Depth = 90% pachymetry on incision location Length = 40° to 60 ° (<6 D), 65° to 75° (6 to 10 D), 90° (> 10 D) 0.5 mm inside the graft-host junction		Graft	7.46 ± 2.7 (K)	4.77 ± 3.29 (K)	36 (K)	3 rejections, 9 overcorrections (24%)
Fares et al., 2013 [66]	26	Nonrandomized prospective study	Manual relaxing incision with compression suture Depth = 80% CCT Length = 45° (6 D to 9 D) to 60° (>9 D) 7 mm zone diameter		Interface	9.66 ± 2.9 (R)	4.37 ± 2.53 (R)	58.4 (R)	1 perforation
Cleary et al., 2013 [42]	6	Prospective noncomparative interventional case series	FS beveled Bevel angle of 135° Depth = 65% to 75% CCT Length = 60° to 90° 0.4 mm inside the graft margin		Graft	9.8 ± 2.9 (K)	4.5 ± 3.2 (K)	57 (K)	None
Loriaut et al., 2015 [67]	20	Retrospective case series	FS Depth = 75% thinnest pachymetry Length = 60° to 80° 6 to 6.8 mm zone diameter (Hanna nomogram [38])		Graft	9.45 ± 2.97 (K)	4.64 ± 2.79 (K)	50.9 (K)	10 overcorrections
Al Sabaani et al., 2016 [41]	52	Retrospective non-comparative interventional study	FS Depth = 75% - 85% thinnest pachymetry Length = 50° to 70° (Nordan nomogram [41]) 0.5-0.7 mm within the graft-host junction		Graft	7.15 ± 1.32 (R)	5.19 ± 2.25 (R)	27.4 (R)	3 perforations, 12 overcorrections
Hashemian et al., 2017 [68]	23	Prospective interventional case series	FS Depth = 85% thinnest pachymetry Length = 40° to 90° (Wu [69] and Kumar [65] nomogram) 1 mm within the graft-host junction		Graft	7.79 ± 2.64 (R)	3.69 ± 2.25 (R)	52.6 (R)	2 perforations

*CCT* = central corneal thickness; *FS* = femtosecond laser assisted

Arcuate keratotomy can be useful as it creates minimal surface disruption, can treat nonorthogonal astigmatism, and yields rapid visual recovery. This technique ensures an optical zone of constant diameter and homogeneity of depth over the entire length of the incision. Typically, post-PK keratotomies are done to a depth of 75% to 80% corneal thickness, and incisions are made at an optical zone 1.0 mm central to the graft-host junction.

Three different incision locations were described: graft, graft-host interface or host cornea. Refractive or keratometric pre-operative cylinder was between 11 D and 6.1 D, and an overall reduction ranging from 30 to 72% was observed, with manual or femtosecond-assisted techniques. Most of the patients with astigmatism higher than 6 D had residual cylinder less or equal to 3 D, which can be treated by laser excimer ablation, or secondary IOL implantation.

In the reported studies, manual keratotomy seems to have more complications (in particular wound dehiscence) and poorer predictability than FSAK. However, all studies had a high variability in outcomes despite the use of a standardized method of surgery. The unpredictability seems to be correlated with many variables such as the value of the initial cylinder, individual variability, alignment.

Individual variability responsible for limited predictability is related to the variability in the distribution of compressive forces and biomechanical constraints in the corneal graft. Therefore, arcuate keratotomy after PK necessitates its own nomogram, as the fibrotic rim created at the graft-host junction has a different tension than the natural limbus.

Under-correction and inter-individual variability can be due to misalignment. This could be addressed by preoperative limbal marking but the direct visualization of the limbus can be masked by the suction ring during the procedure. Also, a control of cyclotorsion can be useful in this case. Femtosecond provides more depth accuracy than manual keratotomy as we have a pre-operative view of the cornea thickness thanks to the OCT.

Unsuccessful correction and overcorrection can be explained by the pattern of irregular astigmatism (the flat and steep meridians are usually not orthogonal, with asymmetric power distribution), which leads to unpredictable correction. Some authors hypothesized that using topography-guided placement of relaxing incision can produce more predictable results, as shown by vector analysis using Alpin’s methods [39, 50, 51].

Unpredictability can also be explained by the inherent dynamic instability of such incisions over time.

It is possible that a smaller arc length would achieve the intended effect on astigmatism or increasing the angular length and reducing the optical zone diameter of the incisions as Wilkins et al. [35] reported.

A refinement in the treatment nomogram for femtosecond laser-assisted AK for high astigmatism after PK remains a major issue. Further prospective studies with different length, depth and width correlated with the pre-operative cylinder and optical zone should be performed to define a new nomogram with a higher predictability.

### Complications

Manual procedures have some disadvantages: poor predictability, overcorrection, corneal perforation, wound dehiscence, and instability of corneal topography [24]. In manual procedures, depth is usually set to 75–85% depth of central corneal thickness (CCT), but pre-operative visualization of the corneal thickness is not possible thereby increasing the risk of perforation and overcorrection due to deep incisions. Deep incisions are also associated with wound dehiscence. The suture of the keratotomy can reduce dehiscence and overcorrection, and can be used to treat micro-perforation. Poor predictability with manual procedures is due to the absence of standardized nomograms correlating the amount of keratometric astigmatism with the extension of the incisions. Nomogram for congenital astigmatism cannot be applied for post-PK astigmatism [52], so newer and more precise nomogram should be studied.

FSAK is safer and more predictable than manual procedures, but complications have also been described (Table 1). Overcorrection or wound gaping can be managed with compressive sutures. Infections are rare but possible, keratitis and one case of endophthalmitis have been reported. Topical post-operative antibioprophylaxis should be sufficient [53]. Graft rejections have also been reported, but topical corticosteroid for 1 month after the surgery can be administered. Corneal perforation can occur, even with per-operative OCT [54], but it is a very rare complication.

## Conclusion

No standard surgical procedure for performing arcuate keratotomies for post-penetrating keratoplasties astigmatism exists, due to its unpredictability. However, all the studies show that patients with astigmatism higher than 6 D had residual cylinder less than or equal to 3 D, which can be further treated by laser excimer ablation or secondary IOL implantation.
